# Different patterns of cortical maturation before and after 38 weeks gestational age demonstrated by diffusion MRI *in vivo*

**DOI:** 10.1016/j.neuroimage.2018.05.046

**Published:** 2019-01-15

**Authors:** Dafnis Batalle, Jonathan O'Muircheartaigh, Antonios Makropoulos, Christopher J. Kelly, Ralica Dimitrova, Emer J. Hughes, Joseph V. Hajnal, Hui Zhang, Daniel C. Alexander, A. David Edwards, Serena J. Counsell

**Affiliations:** a Centre for the Developing Brain, School of Biomedical Engineering & Imaging Sciences, King's College London, SE1 7EH, London, United Kingdom; b Department of Forensic and Neurodevelopmental Sciences & Department of Neuroimaging, Institute of Psychiatry, Psychology and Neuroscience, King's College London, SE5 8AF, London, United Kingdom; c Biomedical Image Analysis Group, Imperial College London, United Kingdom; d Department of Computer Science & Centre for Medical Image Computing, University College London, United Kingdom

**Keywords:** Cerebral cortex, Preterm, Newborn, NODDI, Prematurity, Development, dMRI, diffusion MRI, FA, fractional anisotropy, FDR, false discovery rate, GA, gestational age, MRI, Magnetic resonance imaging, NDI, neurite density index, NODDI, neurite orientation dispersion and density imaging, ODI, orientation dispersion index, PMA, post menstrual age

## Abstract

Human cortical development during the third trimester is characterised by macro- and microstructural changes which are reflected in alterations in diffusion MRI (dMRI) measures, with significant decreases in cortical mean diffusivity (MD) and fractional anisotropy (FA). This has been interpreted as reflecting increased cellular density and dendritic arborisation. However, the fall in FA stops abruptly at 38 weeks post-menstrual age (PMA), and then tends to plateau, while MD continues to fall, suggesting a more complex picture and raising the hypothesis that after this age development is dominated by continuing increase in neural and organelle density rather than alterations in the geometry of dendritic trees. To test this, we used neurite orientation dispersion and density imaging (NODDI), acquiring multi-shell, high angular resolution dMRI and measures of cortical volume and mean curvature in 99 preterm infants scanned between 25 and 47 weeks PMA. We predicted that increased neurite and organelle density would be reflected in increases in neurite density index (NDI), while a relatively unchanging geometrical structure would be associated with constant orientation dispersion index (ODI). As dendritic arborisation is likely to be one of the drivers of gyrification, we also predicted that measures of cortical volume and curvature would correlate with ODI and show slower growth after 38 weeks.

We observed a decrease of MD throughout the period, while cortical FA decreased from 25 to 38 weeks PMA and then increased. ODI increased up to 38 weeks and then plateaued, while NDI rose after 38 weeks. The evolution of ODI correlated with cortical volume and curvature. Regional analysis of cortical microstructure revealed a heterogenous pattern with increases in FA and NDI after 38 weeks confined to primary motor and sensory regions. These results support the interpretation that cortical development between 25 and 38 weeks PMA shows a predominant increase in dendritic arborisation and neurite growth, while between 38 and 47 weeks PMA it is dominated by increasing cellular and organelle density.

## Highlights

• DTI and NODDI cortical measures between 25 and 47 weeks GA
• Early cortical changes consistent with dendritic arborisation and neurite growth
• After 38 weeks cortical changes consistent with increasing cellular density
• Cortical curvature evolves in parallel with dendritic arborisation

## Graphical abstract

**Figure:**
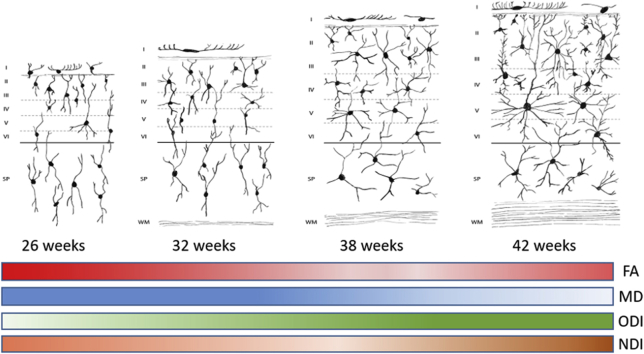

## 1 Introduction

The last trimester of pregnancy and early perinatal period are associated with rapid cortical development including dendritic growth from cortical neurons, in-growth of thalamo-cortical afferents, synapse formation and proliferation of glial cells (Kostovic and Jovanov-Milosevic, 2006). These changes in cell structure and number are accompanied by morphological changes, including the development of cortical gyrification. High-resolution magnetic resonance imaging (MRI) enables the measurement of cortical macrostructural development, including increases in gyrification and cortical grey matter volume, in term-born and preterm infants (Ajayi-Obe et al., 2000; Deipolyi et al., 2005; Dubois et al., 2008b; Makropoulos et al., 2016) and these measures can be related to perinatal risk factors and subsequent outcome (Dubois et al., 2008a; Kapellou et al., 2006; Kaukola et al., 2009; Rathbone et al., 2011). Longitudinal animal studies show that the regional and temporal development of cortical curvature, but not expansion of cortical surface area, occurs in parallel with microstructural maturation, supporting the hypothesis that cortical gyrification is related to microstructural cortical development (Wang et al., 2017).

Diffusion MRI (dMRI) characterises water molecular motion in tissue and provides information on microscopic cortical development. Diffusion Tensor Imaging (DTI) has been used previously in human preterm infants to characterise cortical mean diffusivity (MD), the average displacement of water molecules in tissue, and fractional anisotropy (FA), a measure of the directional dependence of water molecular motion. Both MD and FA values decrease in cortical grey matter from 26 weeks gestation (Ball et al., 2013; McKinstry et al., 2002; Vinall et al., 2013). The elevated anisotropy observed in the preterm period appears to be dependent on diffusivity along the principal direction of the diffusion tensor (axial diffusivity) which is aligned perpendicularly to the pial surface of the brain (McKinstry et al., 2002). In the early third trimester, cortical cytoarchitecture is dominated by neurons migrating towards the cortex along radially organised glial fibres (Rakic, 2003). With increasing maturation, dendritic growth from cell bodies, in-growth of thalamo-cortical afferents, the formation of synapses and proliferation of glial cells produce a complex multidirectional microstructural environment, resulting in a decrease in anisotropy and diffusivity. The changes in cortical anisotropy are regionally specific, with decreases in primary somatosensory regions observed prior to those in the frontal cortex (Ball et al., 2013). These observations are supported by animal studies which have shown that decreasing cortical FA is associated with increased dendritic arborisation and complexity whereas decreases in MD probably reflect increasing cellular and organelle density (Dean et al., 2013; Sizonenko et al., 2007).

However, although MD continues to fall until after the normal time of birth at 40 weeks post-menstrual age (PMA), the fall in FA abruptly stops at 38 weeks PMA and then appears to plateau (Ball et al., 2013; McKinstry et al., 2002). This suggests a more complex picture and raises the hypothesis that, after this age, development is dominated less by alterations in the geometry of dendritic trees and more by increased cellular and organelle density associated with an increase in the number of differentiated neurons and the onset of cortical myelination (Deoni et al., 2015; Takashima et al., 1980).

New multi-compartment dMRI models, such as neurite orientation dispersion and density imaging (NODDI) (Zhang et al., 2012), provide measures that enable this hypothesis to be tested by modelling measured diffusion signal in three compartments: intra-neurite, extra-neurite and free water. NODDI offers additional information to that obtained from DTI models and allows microstructural features to be determined, including neurite density index (NDI) and orientation dispersion index (ODI), which are correlated with histological measures of neurite geometrical complexity (Grussu et al., 2017). Using NODDI to characterise cortical features during development, a previous small sample study reported an increase in cortical ODI alongside a decrease in FA (Eaton-Rosen et al., 2015).

In the present study we used multi-shell, high angular resolution dMRI and NODDI, combined with measures of cortical volume and mean curvature obtained from high spatial resolution structural MRI to address the hypothesis that cortical maturation is dominated by the geometric effects of dendritic arborisation until 38 weeks, and by increasing cellular and organelle density after this period. We predicted that increased neurite and organelle density would be reflected in increases in NDI, while a relatively unchanging geometrical structure would be associated with constant ODI. As dendritic arborisation is likely to be one of the drivers of cortical gyrification, we also predicted that measures of cortical volume and curvature would correlate with ODI and show slower growth after 38 weeks.

## 2 Methods

**Fig. 1:**
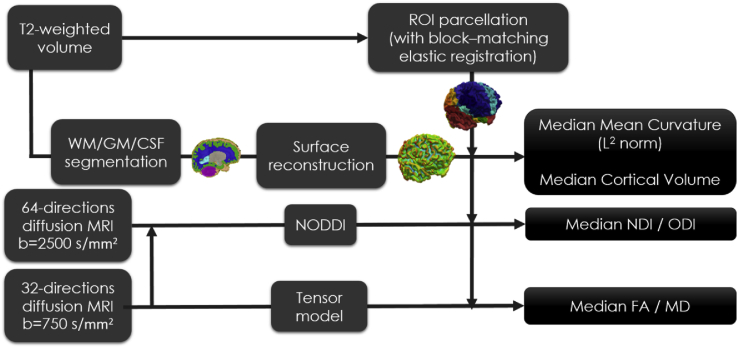
Methodological scheme. White matter (WM), grey matter (GM) and cerebrospinal fluid (CSF) are segmented from T2-weighted volume, as well as regional parcellation and surface reconstruction. Indices of neurite density (NDI) and orientation dispersion (ODI) are obtained from NODDI fitting of multi-shell diffusion MRI. Fractional anisotropy (FA) and mean diffusivity (MD) are obtained from shell at b = 750 s/mm^2^.

See Fig. 1 for a scheme of the methodology used to extract macro- and microstructural information from anatomical and diffusion MRI acquisitions in each subject.

### 2.1 Subjects

The National Research Ethics Committee gave approval for MR imaging (09/H0707/98 and 12/LO/1247) and written parental consent was obtained prior to MRI. Infants were recruited from the neonatal intensive care unit or post-natal ward as part of a research study assessing preterm brain development. The inclusion criteria for this study were gestational age (GA) at birth below 37 weeks and MR imaging without motion artefacts, performed ≤48 weeks PMA. Exclusion criteria were evidence of major focal lesions, including periventricular leukomalacia or haemorrhagic parenchymal infarction, congenital malformation or metal implants. We studied 99 preterm infants (60 male) born between 24 and 37 (median 31) weeks GA and scanned between 25 and 47 (median 40) weeks PMA. Eight infants were scanned twice. MR images were reviewed by an experienced perinatal neuroradiologist. The perinatal characteristics of the infants are described in Table 1.

**Table 1:** Perinatal clinical characteristics of the infants.

Clinical Characteristics	
Median (range) GA at birth (weeks)	31.29 (24–36.71)
Median (range) birthweight (kg)	1.35 (0.605–3.545)
Median (range) PMA at scan (weeks)	39.86 (25.43–47.14)
Number (%) male	60 (60%)
Number of infants (%) with necrotising enterocolitis	7 (7%)
Number (%) of infants Small for Gestational Age^a^	30 (30%)
Median (range) days requiring respiratory support (mechanical ventilation, continuous positive airway pressure and supplementary oxygen)	3 (0–142)

a <10th weight percentile.

### 2.2 MRI acquisition

**Supplementary Fig. S1:**
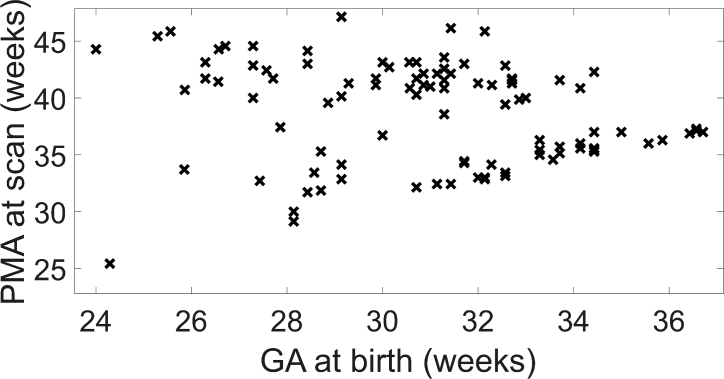
Distribution of postmenstrual age (PMA) at scan according to gestational age (GA) at birth.

MR imaging was performed on a 3 T Philips Achieva system (Best, The Netherlands) sited on the neonatal intensive care unit. 3D MPRAGE (repetition time (TR) = 17 ms, echo time (TE) = 4.6 ms, flip angle 13°, voxel size: 0.82 × 0.93 × 0.5 mm) and T2 weighted fast spin echo (TR = 14473 ms, TE = 160 ms, flip angle 90°, slice thickness 2 mm with 1 mm overlapping slices, in-plane resolution 1.15 × 1.15 mm) were obtained. dMRI data were acquired at 2 mm isotropic resolution with SENSE factor of 2 in 2 shells, acquisition matrix 112 × 110, field of view (FoV) 224 × 224 mm^2^; 64 non-collinear directions with a b-value of 2500 s/mm^2^, 4 non-diffusion weighted images (b = 0) with TR 9093 ms and TE 62 ms; and 32 non-collinear directions with a b-value of 750 s/mm^2^, 1 non-diffusion weighted image (b = 0) with TR 7856 ms and TE 49 ms. Data for 51 infants were acquired with an 8 channel head coil and data for 48 infants (8 scanned twice, leading to 56 acquisitions) with a 32 channel head coil. See in Supplementary Fig. S1 the distribution of age at birth versus age at scan.

All examinations were supervised by a paediatrician experienced in MR imaging. Parents of infants over 36 weeks PMA were offered sedation for their child, oral chloral hydrate (25–50 mg/kg), prior to scanning and 46 infants were sedated for imaging. Pulse oximetry, temperature and electrocardiography data were monitored. Earplugs moulded from a silicone-based putty (President Putty, Coltene, Whaldent, Mahwah, NJ, USA) placed in the external auditory meatus and neonatal earmuffs (MiniMuffs, Natus Medical Inc., San Carlos, CA, USA) were used for auditory protection.

### 2.3 Parcellation and extraction of macrostructural features

**Supplementary Fig. S2:**
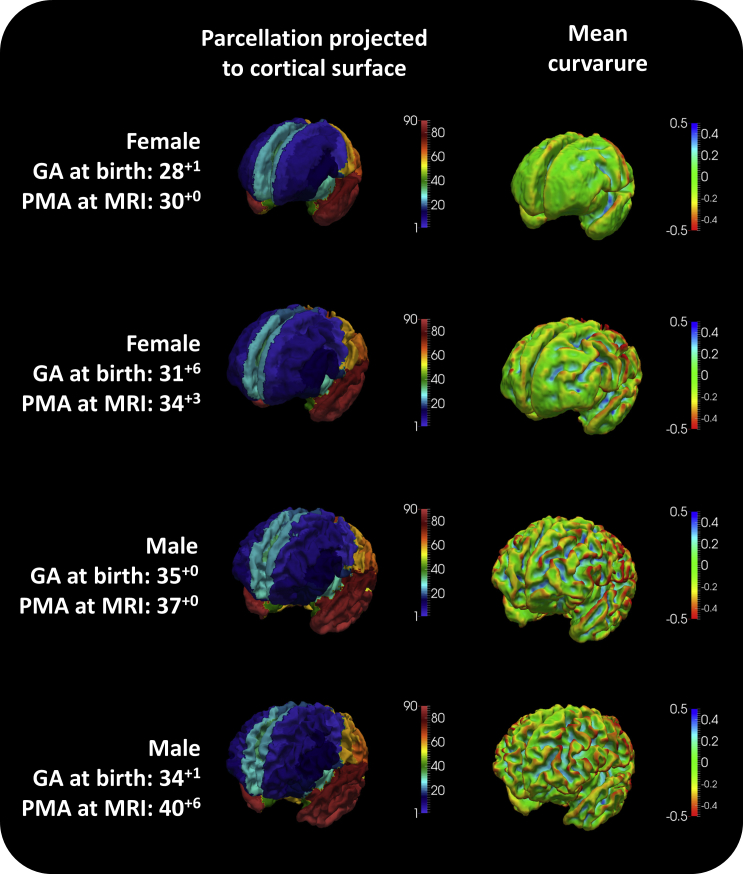
Cortical Surface reconstruction and its overlay with crotical segmentation (AAL atlas) and estimated mean curvature at each vertex in four representative subjects.

T2-weighted brain volumes were bias corrected (Tustison et al., 2010), skull stripped and the tissue segmented into white matter, cortical grey matter, deep grey matter, cerebrospinal fluid and cerebellum using a neonatal-specific segmentation algorithm (Makropoulos et al., 2014). Parcellation into cortical regions was performed with a block matching non-linear registration (Tristan-Vega and Arribas, 2007) of a version of the standard anatomical automatic labelling (AAL) atlas (Tzourio-Mazoyer et al., 2002), specifically adapted to the neonatal brain (Shi et al., 2011). Parcellation of the atlas was propagated into each subject's native T2 space following the non-linear registration previously calculated and a nearest neighbour propagation. Sub-cortical (caudate, putamen, pallidum and thalami) and cerebellar regions were not considered, resulting in a total of 82 regions in each subjects' native space. The cortical surface of each subject was reconstructed based on the tissue parcellation in T2 native space using a methodology specifically developed for the neonatal brain (Makropoulos et al., 2016), and measures of cortical curvature (mean curvature *L*^2^ norm) were obtained for each surface vertex. Median absolute mean curvature was obtained for the total cortical grey matter and each cortical region. See Supplementary Fig. S2 for examples of cortical parcellation projected on the cortical surface and estimation of mean cortical curvature at different ages.

### 2.4 Diffusion MRI pre-processing

Pre-processing of diffusion MRI data was performed following a previously described protocol (Batalle et al., 2017). Briefly, diffusion MRI volumes were first visually inspected in order to detect data with motion artefacts, and exclude them from further analysis. All subjects included in the study had at most 8 (median 2, range 0–8) gradient directions excluded from the higher shell, and at most 6 (median 1, range 0–6) gradient directions excluded from the lower shell. Volumes were first corrected for EPI phase encoding distortions, eddy-induced distortions and subject movements by means of FSL5.0 topup-eddy algorithm (Andersson et al., 2003; Andersson and Sotiropoulos, 2015), using T2 volume rigidly registered to b = 0 maps and assuming a bandwidth of zero (no phase-encoding). This process was performed separately for the two acquired shells and their corresponding *b* = 0 volumes, and then the lower shell was rigidly registered to the averaged *b* = 0 volumes acquired with the higher shell. Gradient directions were rotated accordingly. Finally, T2-weighted volumes were rigidly registered to the average b = 0 volume of each subject. This registration was used to transform the white matter/grey matter segmentations and previously computed parcellations of cortical regions to the native diffusion space of each subject. All rigid registrations were performed with IRTK software (Studholme et al., 1999).

### 2.5 Extraction of microstructural features

The diffusion tensor was fitted to the dMRI data with *MRTrix3* (Tournier et al., 2012) using only the lower shell (b = 750 s/mm^2^) and both FA and MD were obtained at every voxel.

The NODDI toolbox (Zhang et al., 2012) was used to obtain maps of estimated NDI and ODI for each subject. Briefly, NODDI models diffusion in each voxel as three independent compartments: intra-neurite, extra-neurite and free water compartment. NODDI describes the normalised diffusion signal in each of these three compartments as (Zhang et al., 2012):

$$A=\left(1-V_{iso}\right)\left(V_{in}A_{in}+\left(1-V_{in}\right)A_{en}\right)+V_{iso}A_{iso}$$

Where $A_{in}$ and $V_{in}$ represent the normalised signal and volume fraction of intra-neurite compartment; $A_{en}$ represents the normalised signal of extra-neurite compartment and $A_{iso}$ and $V_{iso}$ represent the normalised signal and volume fraction of the free water compartment.

The intra-neurite compartment models the space occupied by neurites, and is represented by a set of ‘sticks’. The distribution of sticks is modelled as a Watson distribution with free parameter κ (ranging from 0 to infinite). ODI is derived from fitted κ simply as $ODI=\frac{2}{\pi }\left(\tan^{-1}1/\kappa \right)$, allowing us to obtain a parameter ranging from 0 to 1 describing orientation distribution of intra-neurite compartment. We refer to $V_{in}$ as NDI since it represents the density of neurites outside of the free-water compartment. Extra-neurite compartment models water diffusing on the space around neurites, capturing the restricted diffusion of water orthogonally to neurites and unhindered along them. Finally, a free water compartment models diffusion of free water (i.e., CSF). See (Zhang et al., 2012) for a detailed formulation of the model. Importantly, note that NODDI assumes fixed compartment diffusivities, which are optimised for the adult brain, but might not be the best fit for our sample (Jelescu et al., 2015), and could bias the estimation of certain parameters. However, since there are no reference values available for the studied age, we used default values provided by the NODDI toolbox (see *Discussion* section).

**Supplementary Fig. S3:**
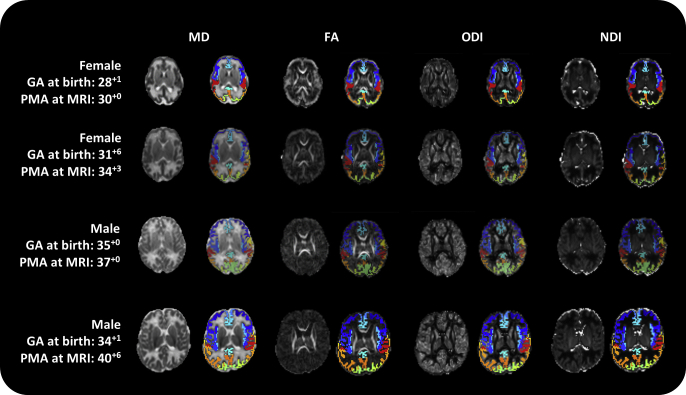
Regional grey matter parcellation overlayed on MD, FA, ODI and NDI in 4 representative subjects.

In order to take into account the difference in diffusion signal due to different TE/TR in the two diffusion shells, we normalised each shell diffusion volumes by the b = 0 corresponding to each shell. NODDI grid search starting points were modified in order to better fit neonatal data by lowering the range of values considered as the fraction of the intra-neurite space from 0 to 1 to 0–0.3 as established by Kunz et al. (2014) (included as part of NODDI toolbox as “invivopreterm” tissue type). In addition, we used AMICO, a linearised version of NODDI (Daducci et al., 2015), to provide initialisation parameters in the voxels where the initial fitting didn't converged before repeating NODDI toolbox fitting in those voxels. Median values of FA, MD, NDI and ODI were obtained for grey matter tissue type and for the intersection of grey matter and the cortical regions previously parcellated and down-sampled to each subject native diffusion space. In order to minimise partial volume effects, an additional threshold of f_iso_<0.5 was used to consider a voxel as part of the assessed cortical regions. See Supplementary Fig. S3 for examples of grey matter cortical regions assessed in diffusion space at different ages.

### 2.6 Statistical analysis

The association between cortical macrostructural and microstructural features and PMA at scan was assessed by Spearman's partial correlations controlling for sex, birthweight below the 10th centile, respiratory support, head coil (8 or 32-channels) and GA at birth. When repeated measures were present (i.e., when performing correlations across the whole age range, 25–47 weeks PMA, 8 infants were included at 2 time-points), covariates were regressed out using linear mixed-effects models (LME) including a subject dependent random effect for the intercept to account for repeated measures. Region-wise correlations were estimated independently for PMA at scan <38 and ≥ 38 weeks. Independent inflection points for each region were estimated as the breaking point (between 1st and 3rd quartile of PMA distribution, i.e., between 35 and 42 weeks PMA) of a biphasic linear regression. Their corrected Akaike Information Criterion (Hurvich and Tsai, 1989) was compared with a monophasic linear regression. Association between macro- and microstructural features was assessed by Spearman's correlations, while Spearman's partial correlations including PMA as a covariate were also assessed. Region-wise correlations were corrected with a False Discovery Rate (FDR) approach controlling alpha error to 5% per contrast (Benjamini and Yekutieli, 2001) and visualised with the BrainNet Viewer (Xia et al., 2013).

## 3 Results

### 3.1 Whole-brain grey matter tissue analysis

**Fig. 2:**
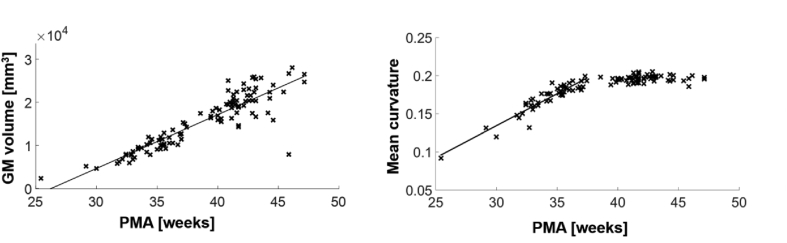
Whole-brain grey matter tissue association between macrostructural characteristics and postmenstrual age at scan. Significant correlations (p < 0.05) were indicated with a regression line.

Total cortical grey matter volume increased significantly with increasing age at scan (ρ = 0.82, p < 0.0001). Mean curvature demonstrated a biphasic relationship with maturity with a significant increase in curvature between 25 and 38 weeks PMA (ρ = 0.78, p < 0.0001), and then no significant change between 38 and 47 weeks PMA (Fig. 2).

**Fig. 3:**
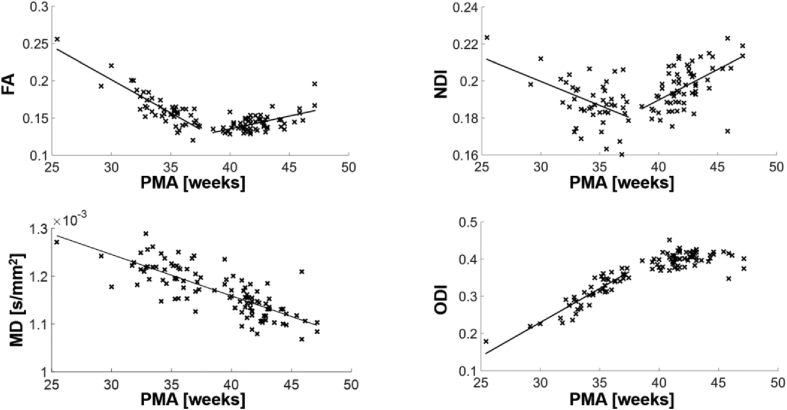
Whole-brain grey matter tissue association between microstructural characteristics and postmenstrual age at scan. Significant correlations (p < 0.05) were indicated with a regression line.

**Supplementary Fig. S4:**
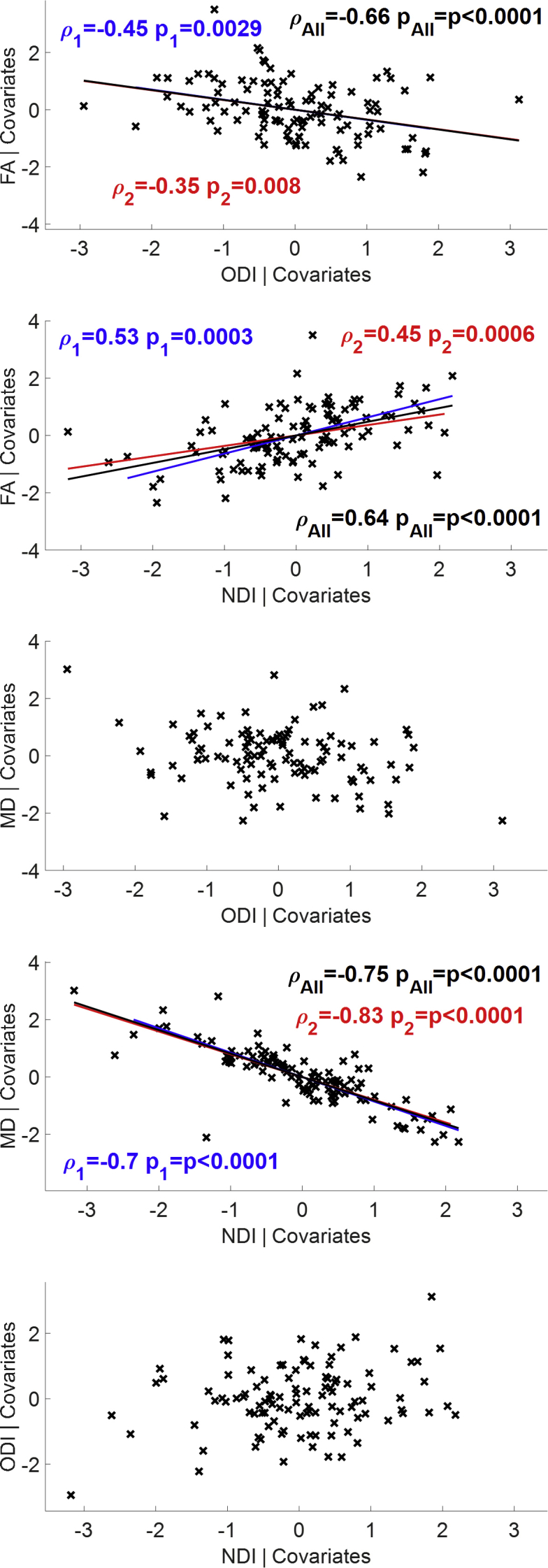
Spearman partial correlations of the residuals of DTI and NODDI features controlling for covariates (including PMA) for whole-brain grey matter tissue. Significant correlations are indicated with regression lines for subjects <38 weeks PMA (*1*, blue), >38 weeks PMA (*2*, red) or the whole sample (*all*, black line).

**Supplementary Fig. S5:**
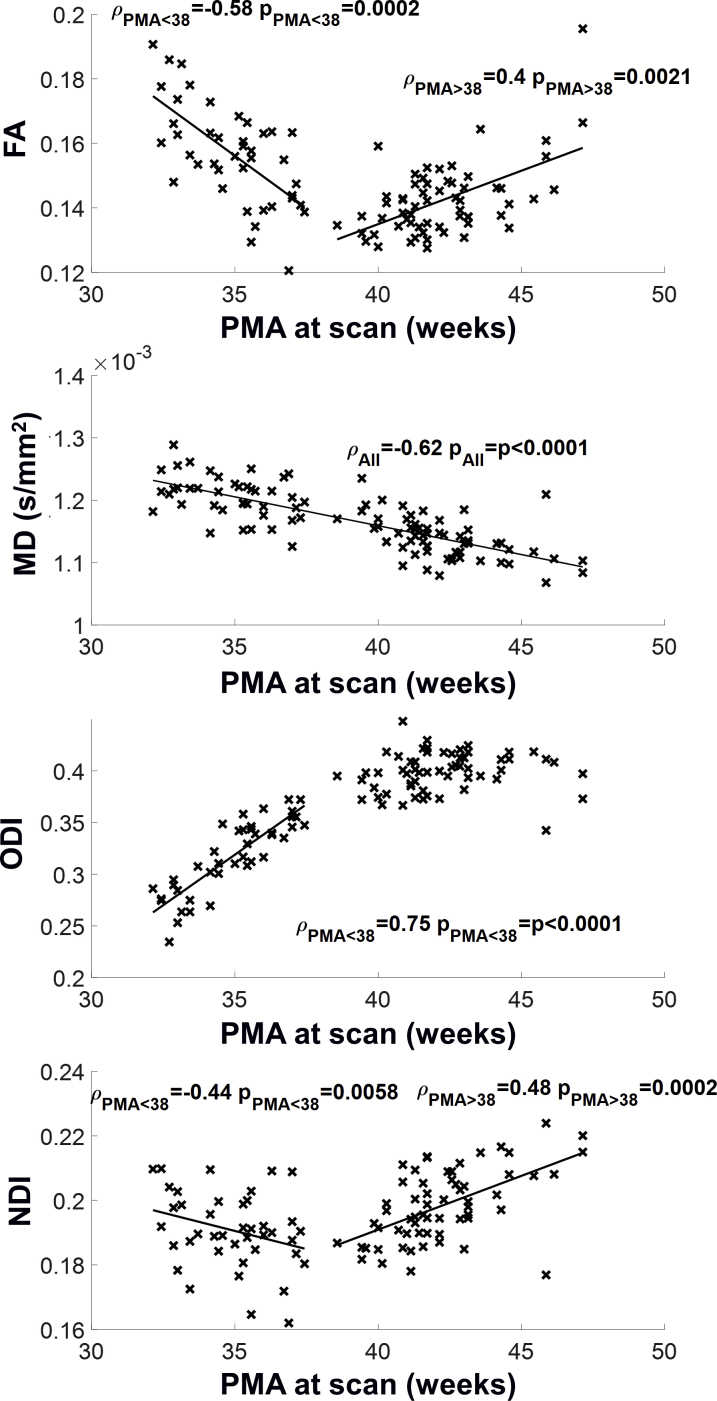
Whole-brain tissue analysis excluding subjects scanned <32 weeks PMA. Significant Spearman partial correlations <38, >38weeks and for the whole sample are highlighted with a regression line.

**Supplementary Fig. S6:**
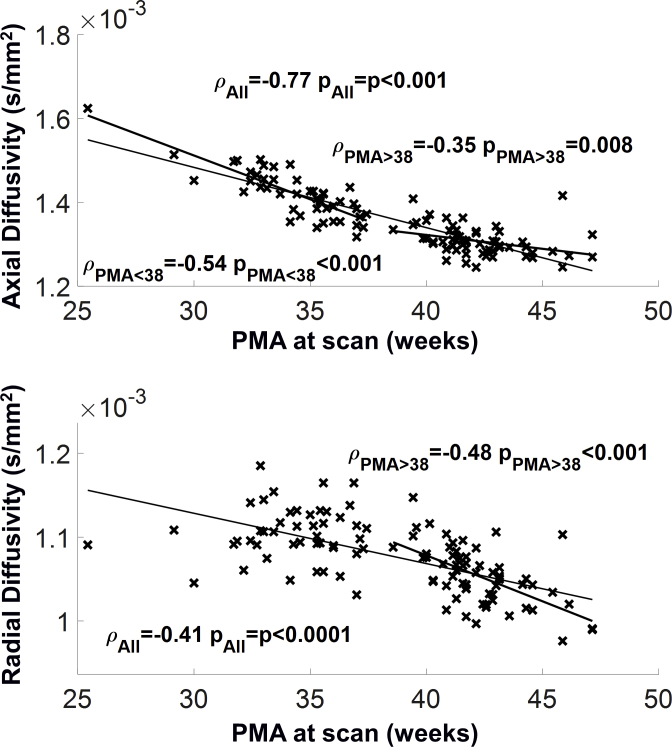
Whole-brain grey matter tissue Spearman partial correlations between Axial and Radial diffusivity and postmenstrual age (PMA) at scan.

Assessing the relationship between cortical microstructural features and increasing PMA revealed a complex pattern of microstructural changes (Fig. 3). FA and NDI decreased significantly between 25 and 38 weeks PMA (ρ = −0.66, p < 0.0001 and ρ = −0.45, p = 0.0027 respectively), and then increased significantly from 38 to 47 weeks PMA (ρ = 0.43, p = 0.0011 and ρ = 0.47, p = 0.0003 respectively). ODI increased significantly between 25 and 38 weeks (ρ = 0.77, p < 0.0001) and then there was little change between 38 and 47 weeks PMA. MD decreased significantly from 25 to 47 weeks PMA (ρ = −0.63 p < 0.0001). Correlations between DTI and NODDI characteristics are demonstrated in Supplementary Fig. S4. Importantly, to ensure that the results are not driven by outliers, we repeated the analysis without subjects scanned before 32 weeks PMA, obtaining very similar results (Supplementary Fig. S5). Radial and axial diffusivity were both negatively correlated with PMA (Supplementary Fig. S6).

### 3.2 Regional analysis

**Fig. 4:**
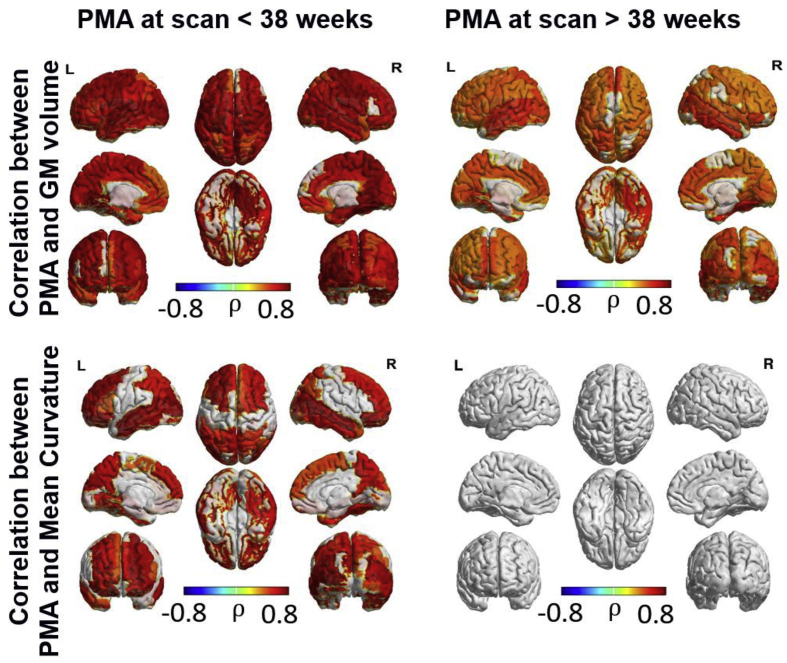
ROI-wise Spearman partial correlation between macrostructural characteristics and postmenstrual age at scan (FDR corrected at 5%).

Region-wise association between macrostructural characteristics and PMA at scan (Fig. 4) showed a widespread increase in cortical grey matter volume throughout the age range studied here, consistent with whole-brain grey matter tissue results. Mean curvature was significantly associated with PMA at scan during the period up to 38 weeks, with the strongest relationship in the frontal and temporal lobes. There was no correlation between curvature and PMA between 38 and 47 weeks PMA.

**Fig. 5:**
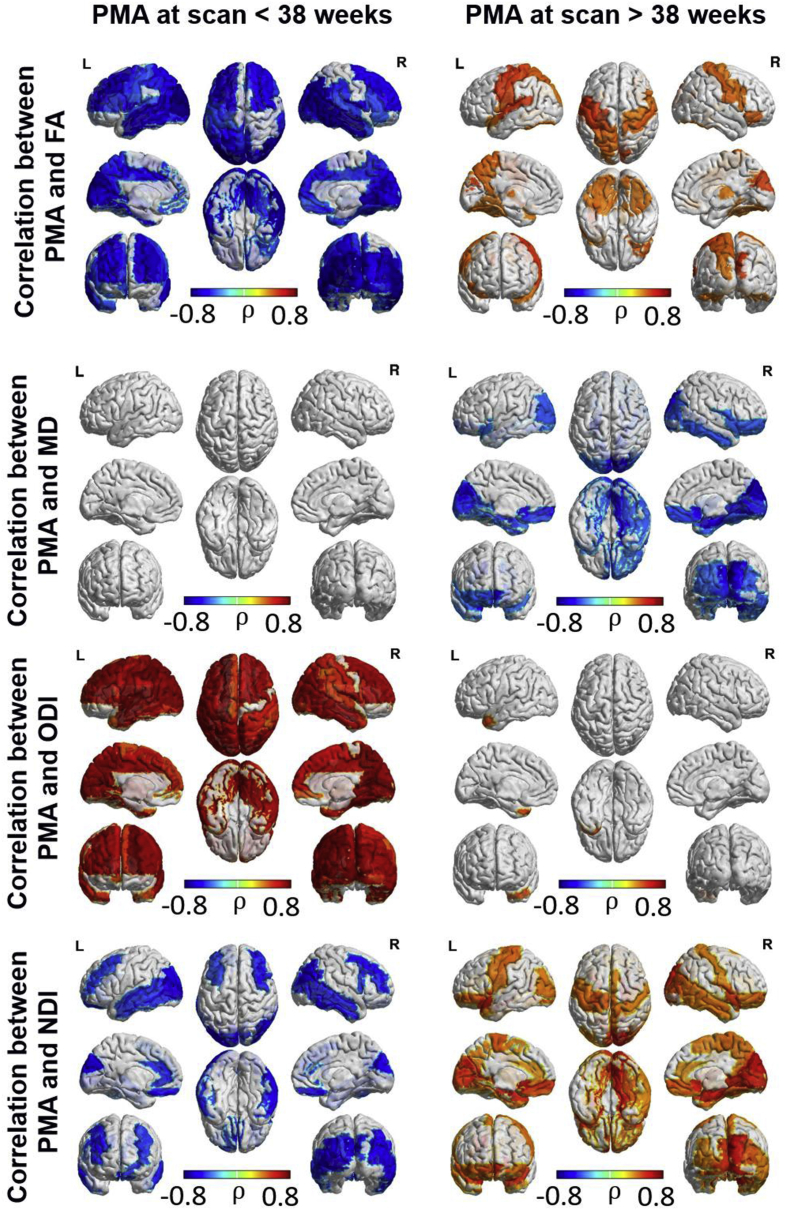
ROI-wise Spearman partial correlation between microstructural characteristics and postmenstrual age at scan (FDR corrected at 5%).

**Supplementary Fig. S7:**
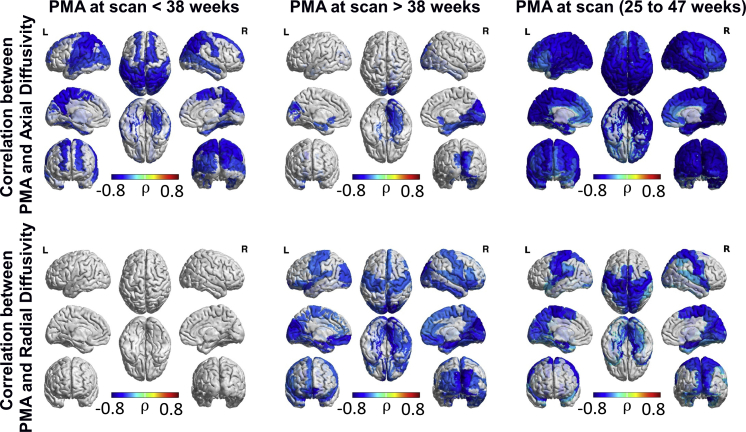
ROI-wise Spearman partial correlation between Axial and Radial diffusivity and postmenstrual age (PMA) at scan (FDR corrected at 5%).

Region-wise assessment of the association between cortical microstructural features and PMA at scan (Fig. 5) showed a heterogeneous regional pattern of maturation. FA was negatively correlated with age at scan in most of the cortex between 25 and 38 weeks PMA but was positively correlated with age at scan in somatosensory regions between 38 and 47 weeks PMA. ODI increased throughout the cortex up to 38 weeks PMA, and then plateaued between 38 and 47 weeks PMA. Interestingly NDI decreased in the middle frontal, cuneus, temporal and occipital gyri between 25 and 38 weeks PMA. NDI increased in somatosensory areas and occipital and orbitofrontal cortices between 38 and 47 weeks PMA. The relationship between MD and maturation did not survive correction for multiple comparisons prior to 38 weeks, but MD decreased significantly in occipital and frontal regions between 38 and 47 weeks PMA. Regional associations between axial and radial diffusivity and PMA are shown in Supplementary Fig. S7.

**Supplementary Fig. S8:**
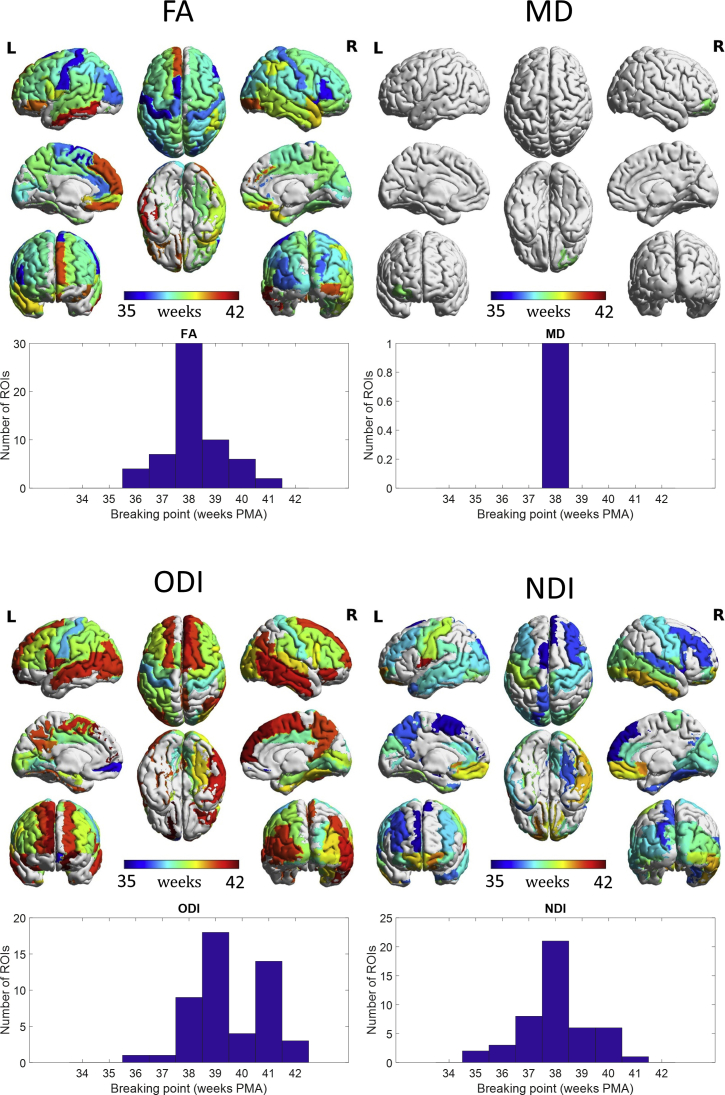
Region-wise biphasic linear regression breaking points (weeks PMA) of DTI and NODDI characteristics, and their histogram. We considered only breaking points between 1st and 3rd quartile of PMA distribution (35-42 weeks) corresponding to a biphasic model with a corrected Akaike Information Criteria (cAIC) inferior to the cAIC of a monophasic linear regression and with a significant (p<0.05) correlation at either side of the breaking point.

**Supplementary Fig. S9:**
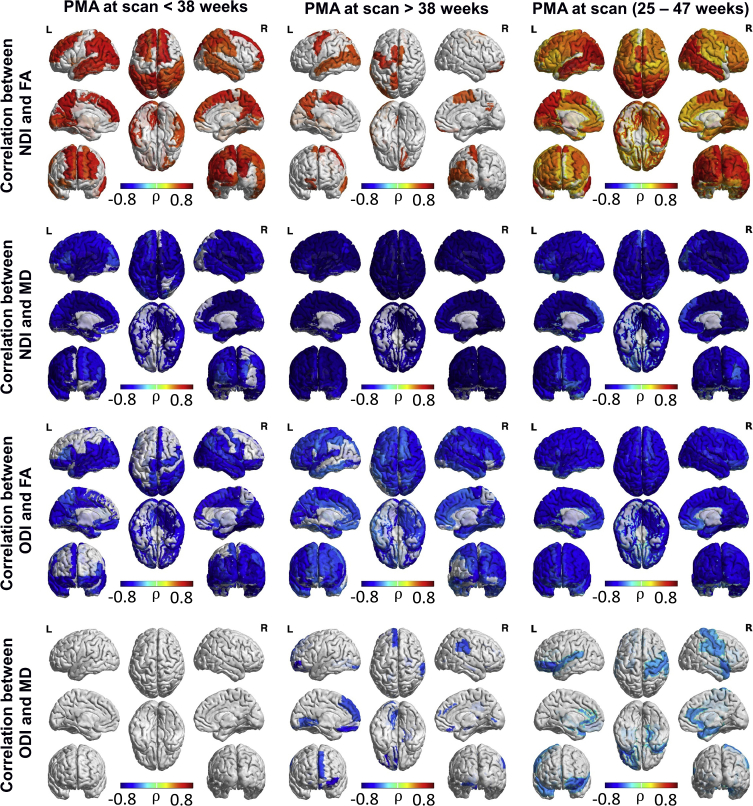
ROI-wise Spearman partial correlation between DTI and NODDI features (FDR corrected at 5%)

Note that although the inflection point for most regions is centred at 38 weeks PMA, there is a certain region-wise variability (characterised in Supplementary Fig. S8 as the breaking point of biphasic linear regression). However, in order to be able to compare the development of all cortical regions and characteristics simultaneously, we show the results using the same inflection point (38 weeks) for all regions and characteristics. See regional relationship between DTI and NODDI characteristics in Supplementary Fig. S9.

### 3.3 Association between micro- and macrostructural features

**Fig. 6:**
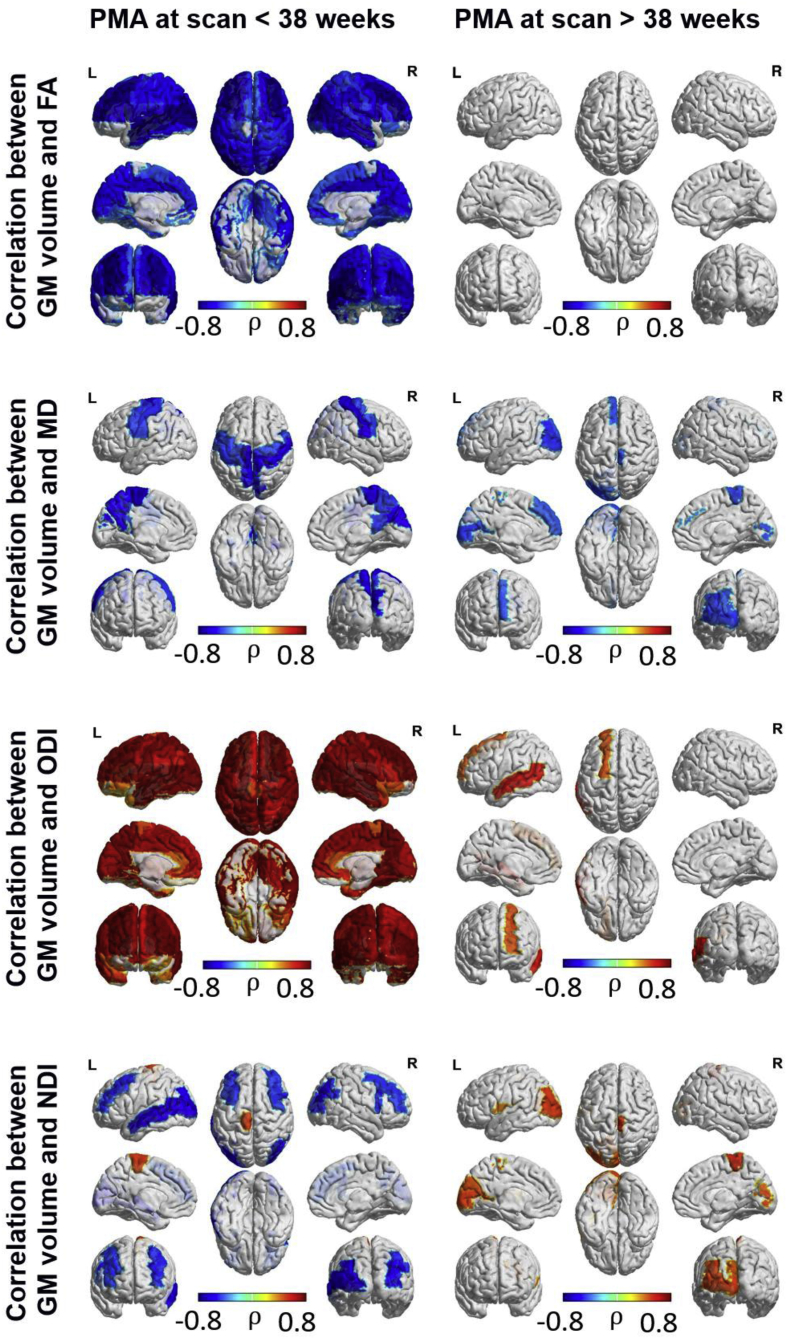
ROI-wise Spearman correlation between grey matter volume and microstructural characteristics. FDR corrected at 5%.

**Fig. 7:**
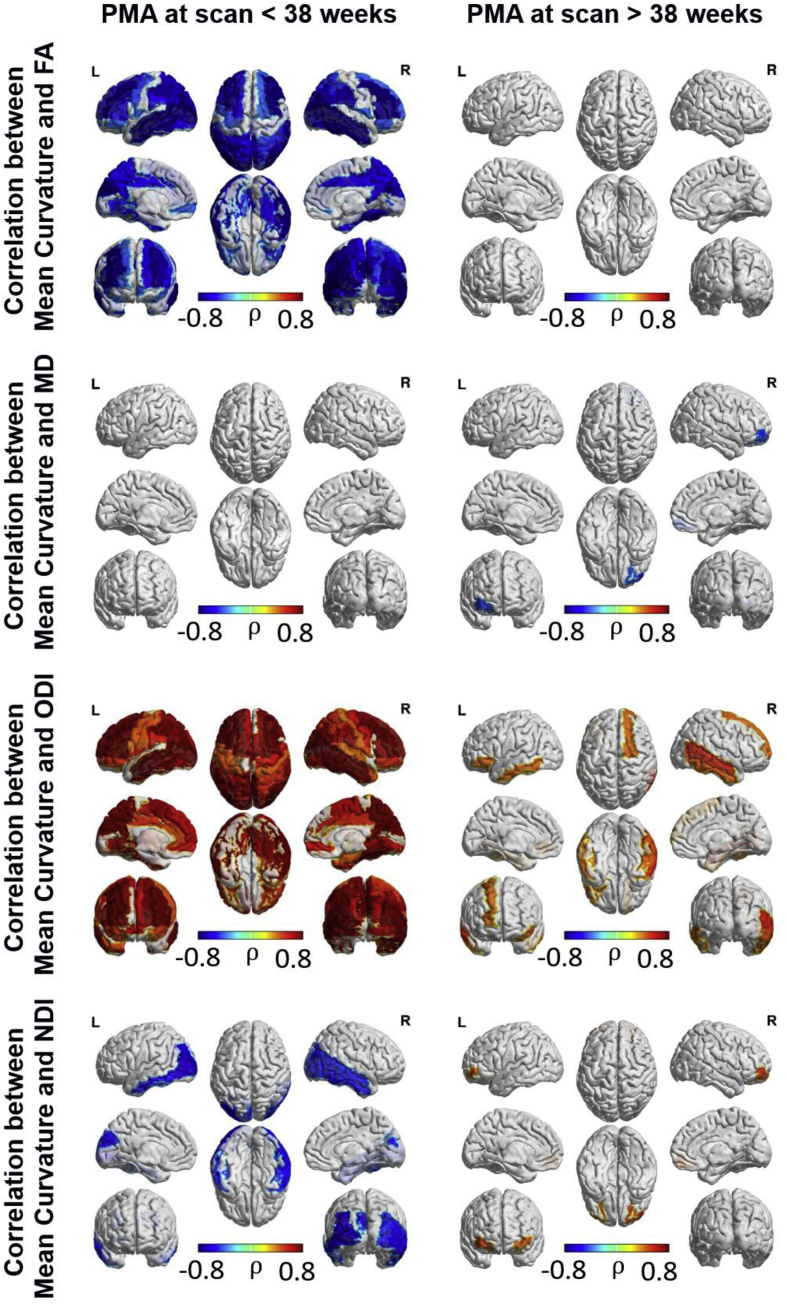
ROI-wise Spearman correlation between mean curvature and microstructural characteristics. FDR corrected at 5%.

Region-wise correlation of microstructural and macrostructural characteristics (see Fig. 6 and Fig. 7) showed that decreasing FA and increasing ODI prior to 38 weeks PMA was correlated with increasing cortical grey matter volume and mean curvature. However, these correlations were not evident after 38 weeks PMA, where cortical grey matter volume was correlated with ODI in only the left frontal superior gyrus and middle temporal gyrus and mean curvature was correlated with ODI in the right superior frontal and middle temporal gyri, left orbitofrontal gyrus and the inferior temporal gyri bilaterally. MD was negatively correlated with cortical grey matter volume in the primary motor and sensory cortices, paracentral gyri, precuneus bilaterally and right cuneus prior to 38 weeks PMA. After 38 weeks PMA, MD was negatively correlated with grey matter volume in the left medial superior frontal gyri, left calcarine and middle and superior occipital gyri, and the right paracentral gyrus.

Of interest, prior to 38 weeks PMA, NDI in bilateral middle frontal and occipital regions and left middle temporal lobe was negatively correlated with grey matter volume, while NDI in the left paracentral lobule showed a positive correlation with grey matter volume. After 38 weeks PMA, NDI was positively correlated with grey matter volume in the right paracentral lobule, and left occipital regions. NDI was negatively correlated with mean curvature in the occipital and temporal regions bilaterally prior to 38 weeks PMA, and positively correlated with mean curvature in the bilateral orbitofrontal gyri after 38 weeks PMA.

**Supplementary Fig. S10:**
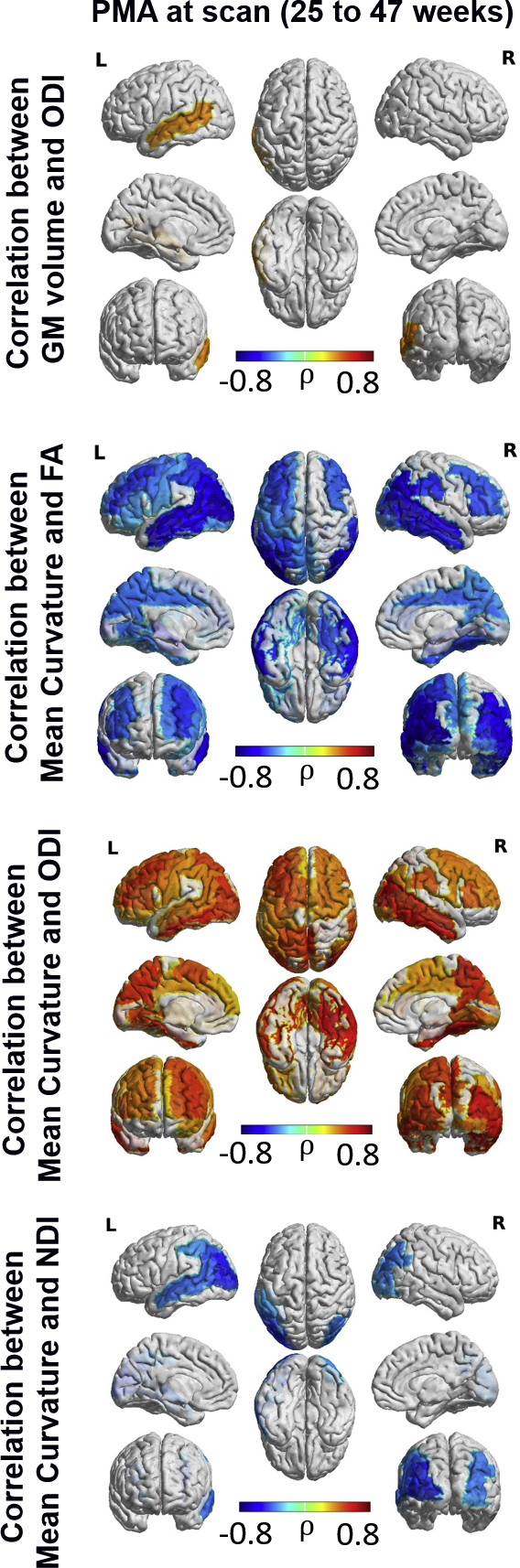
ROI-wise Spearman partial correlation between macrostructural and microstructural features (correcting for all covariates, including PMA at scan, FDR corrected at 5%).

In order to assess the association between micro- and macrostructural characteristics independently of age, we also computed region-wise partial correlations controlling for PMA at MRI in addition to the other covariates (sex, birthweight below the 10th centile, respiratory support, head coil and GA at birth). This analysis revealed a positive correlation between ODI and grey matter volume in left temporal gyrus, and between ODI and mean curvature mainly in frontal and temporal regions (see Supplementary Fig. S10). A negative correlation between mean curvature and FA was also found in temporal regions, as well as in the cingulate gyrus and occipital areas, while mean curvature was negatively correlated with NDI in left temporal gyrus and occipital areas.

## 4 Discussion

**Fig. 8:**
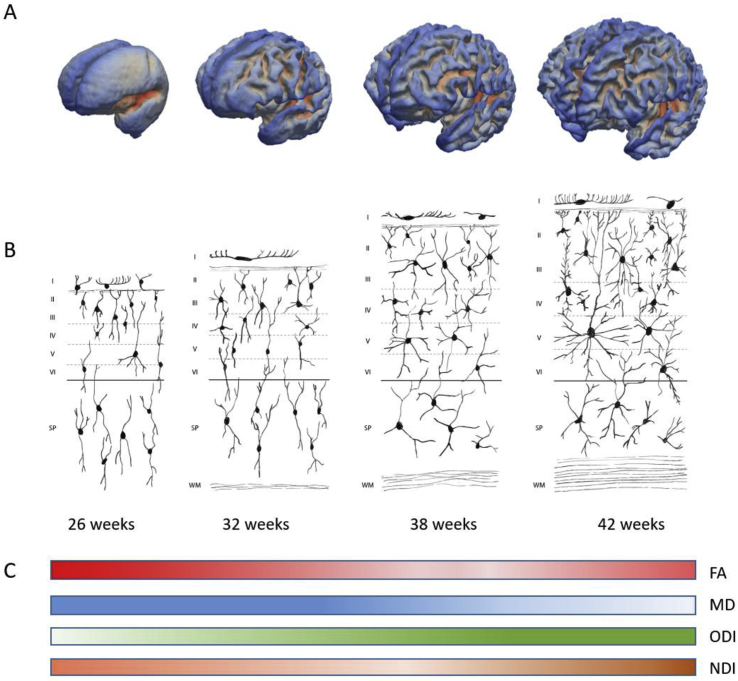
(A) Surface models of the developing brain at 26, 32, 38 and 42 weeks gestational age. (B) Schematic depicting cortical layers with increasing maturation showing cortical and subplate neurons and increasing elaboration of their dendritic spines (adapted from Mrzljak et al., 1988). (C) Schematic of changes in FA (red), MD (blue), ODI (green) and NDI (orange) with increasing gestational age. Increases or decreases in colour intensity represent increasing or decreasing value of the diffusion metric with maturation.

In this study we assessed both macrostructural and microstructural cortical development in the preterm brain between 25 and 47 weeks PMA and observed a biphasic pattern of cortical maturation with steep decreases in FA and increases in ODI prior to 38 weeks, and increased NDI and FA, with no changes in ODI after 38 weeks. Our dMRI findings represent the complex, regionally heterogenous pattern of cortical microstructural and macrostructural development which takes place in the last trimester of pregnancy and which can be studied *in vivo* in preterm infants. Between 25 and 38 weeks gestation, dendritic growth from cell bodies decreases the radial organisation of cortical microstructure, which decreases the principal direction of diffusion, resulting in a decrease in FA. This increasing dendritic arborisation results in an increase in ODI. NDI reduces in some regions between 25 and 38 weeks PMA, probably related to cortical expansion, as described in human autopsy studies (Huttenlocher, 1990). At 38 weeks gestation, the columnar organisation of the cortex is no longer evident and the lateral and radial spread of dendrites results in high ODI. From this age, the increase in dendritic branching has little effect on ODI at a voxel level but increasing neurite density in primary motor, sensory and visual cortex (Huttenlocher, 1990) contributes to an increase in measured NDI from ∼38 weeks. This is accompanied by a decrease in MD. From this age, as ODI remains relatively constant and NDI increases in some regions, measured FA also increases in these regions (see Fig. 8 for a summary of the results and a schematic of the mechanisms described). These findings are consistent with histological studies that show that cortical maturation in the period before normal birth is dominated by increasing geometric complexity associated with dendritic arborisation, and that increased neuronal and organelle density are prevalent from around 38 weeks PMA. We also observed a strong correlation between cortical morphological development and ODI, consistent with a parallel trajectory of arborisation and gyrification maturational processes.

Animal studies allow maturational changes in cortical diffusion measures to be correlated with histology. Decreases in MD and FA have been demonstrated in the developing rat brain between postnatal days 3 and 6 (Sizonenko et al., 2007). This study showed that, while radial organisation was apparent throughout the cortex, with the primary eigenvector perpendicular to the pial surface, this was most apparent in the external cortical layers. With increasing maturation, diffusivity decreased throughout the cortex, but FA values decreased only in the deep cortical layers. These changes in diffusion measures occurred in parallel with a reduction in radial glial scaffolding and increased dendritic density (Sizonenko et al., 2007).

Human autopsy samples show that dendritic arborisation, glial proliferation, neuronal differentiation and synapse formation occurs between 25 weeks and the time of normal birth (Huttenlocher and Courten, 1987; Marin-Padilla, 1992; Mrzljak et al., 1988; Prinz et al., 1997; Rakic, 2003), consistent with our finding of decreasing FA and increasing ODI prior to 38 weeks. We found that ODI values plateaued between 38 and 47 weeks PMA, which is in line with reports that basal (but not apical) dendritic branching is considered complete at 38–40 weeks PMA (Becker et al., 1984; Huttenlocher, 1990; Lu et al., 2013). Previous studies of preterm cortical development have demonstrated reduced FA and MD (Ball et al., 2013; Deipolyi et al., 2005; Eaton-Rosen et al., 2017; McKinstry et al., 2002; Smyser et al., 2016; Vinall et al., 2014) and increased ODI (Eaton-Rosen et al., 2015) up to the time of normal birth. McKinstry et al. (2002) originally described decreased FA with development and proposed that, in the immature cortex, water diffusion is highly anisotropic due to the alignment of apical dendrites and radial glial fibres perpendicular to the cortical surface, restricting water diffusion in a direction parallel to the cortical surface. However, to our knowledge, no studies have reported an increase in FA and NDI in the cortical grey matter after 38 weeks.

Regional analysis revealed that this cortical microstructural maturation was not consistent across the whole of the cortex. While one must take care when extrapolating findings across species, animal studies demonstrate a rostrolateral to caudal/medial gradient in cortical diffusion anisotropy, which appears to mirror patterns of neurogenesis and synapse formation (Huang et al., 2008; Kroenke et al., 2007). In the developing ferret brain, decreases in anisotropy were described in primary cortical areas prior to neighbouring areas, suggesting early innervation by axonal fibres within primary cortical areas prior to other areas (Kroenke et al., 2009). Previous studies in human infants have observed that decreases in diffusion anisotropy occur initially in sensorimotor cortex and later in the frontal lobe (Ball et al., 2013; Deipolyi et al., 2005; Trivedi et al., 2009). In this study, we observed that maturation of primary motor and sensory regions differed to that in association areas, consistent with heterochronicity of cortical myelinogenesis and synaptogenesis described on histology (Flechsig Of Leipsic, 1901; Huttenlocher and Dabholkar, 1997). In particular, after 38 weeks, an increase in FA was observed in primary somatosensory cortices and MD decreased in primary visual areas and inferior frontal lobe. During this period, NDI increased in those regions where we observed increased FA or decreased MD. These findings are consistent with increasing neurite and organelle density with little change in geometrical structure (dendritic branching) after 38 weeks. Indeed, Takashima and colleagues described an increase in the number of differentiated neurons between 20 and 40 weeks gestation, with a marked increase in stellate and other association neurons between 35 and 48 weeks in human visual cortex (Takashima et al., 1980), consistent with our findings of increased NDI at this age. Increases in NDI in pre- and post-central sulcus may also be driven by myelination in this region, as the primary motor and sensory cortices are the first to myelinate (Deoni et al., 2015; Flechsig Of Leipsic, 1901). Of note, FA is intrinsically linked to changes in ODI and NDI (Zhang et al., 2012): increasing geometrical complexity (as measured by increased ODI) will decrease FA, while increased organelle density or myelination (as measured by increased NDI) will increase FA values. Hence, studying ODI and NDI helps elucidate the underlying biological changes associated with alterations in FA and showed that FA increases observed in primary motor and sensory cortices after 38 weeks PMA reflect increases in NDI with no change in ODI (Zhang et al., 2012).

In order to increase our understanding of the relationship between cortical microstructural and macrostructural development, we compared microstructural findings with changes in cortical volume and curvature. Increases in grey matter volume and mean curvature up to 38 weeks PMA were strongly associated with decreases in FA and increases in ODI, highlighting the link between macrostructural cortical maturation and dendritic arborisation that has been described previously in non-human primates (Wang et al., 2017). Similarly, decreased MD was associated with increased volume in primary somatosensory regions. The relationship between NDI and macrostructural measures were less apparent, with only the relationship between mean curvature and volume and decreased NDI in temporal, occipital and some parts of frontal lobe surviving corrections for multiple comparisons. The decrease in NDI in these regions prior to 38 weeks is supported by detailed studies of development of the human visual cortex, which show a decrease in the density of neurons from around 62 × 10^4^ neurons per mm^3^ at 28 weeks GA to 9.6 × 10^4^ neurons per mm^3^ at the time of normal birth (Huttenlocher, 1990). Huttenlocher suggested these reductions in neuronal density were secondary to expansion of the volume of the cortex, which concurs with our findings showing that the rate of increasing volume and curvature in temporal and occipital regions is greater than the rate of increase of cellular and synaptic density as measured by NDI.

**Supplementary Fig. S11:**
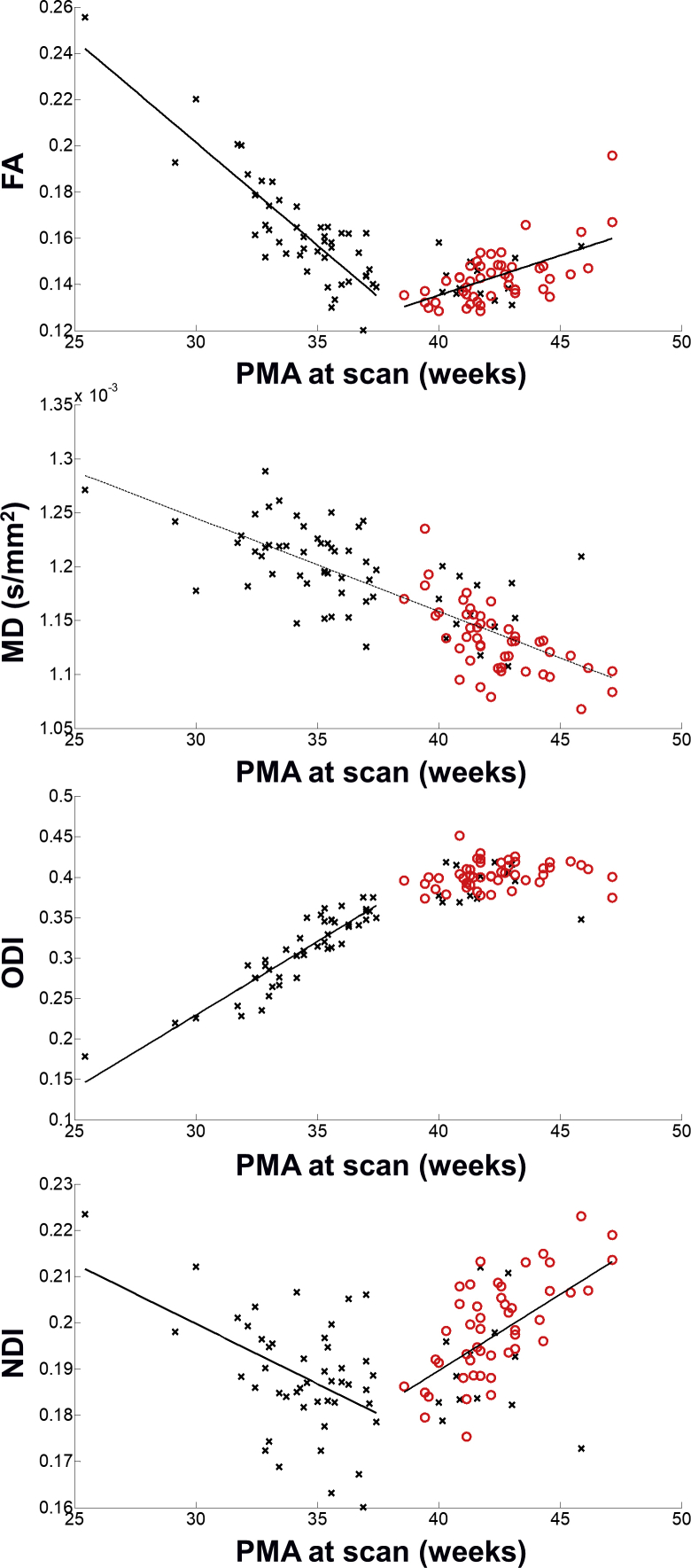
Whole-brain (GM) microstructural characteristics versus age, discriminating for coil type. Note the consistency of results across different coil types. Red circles for 8-channel coil, black crosses for 32-channel coil.

There are a number of limitations with our study: 1) The NODDI model assumes fixed compartment diffusivities rather than considering them a free parameter in the model, which may bias other model parameters. The impact of model assumptions on findings obtained during postnatal brain development have been described previously (Jelescu et al., 2015). However, it has also been shown that current data do not support the freeing of intrinsic diffusivity, as this introduces severe instabilities in fitting, which impact on the estimated model parameters (Jelescu et al., 2016). While several methods have been proposed to stabilise NODDI non-linear fitting (for a review see (Jelescu and Budde, 2017)), validation of these approaches remains challenging. However, NODDI indices are sensitive to underlying biological changes in the brain and are a practical approach to obtain more specific microstructural characteristics than those obtained with DTI parameters in an acceptable acquisition time, which is key when imaging vulnerable preterm infants. Interestingly, NODDI measures have recently been correlated with changes in neurite geometrical configuration assessed with histology in a population with spinal cord multiple sclerosis (Grussu et al., 2017), highlighting the validity of the model indices as proxies of underlying biological changes in microstructure. 2) We applied NODDI to diffusion MRI acquired with different TEs on each shell. While using the same TE for different shells is recommended for prospective studies, in retrospective studies, like ours, this issue has been addressed by first computing the normalised signal attenuation for each shell (relative to b = 0 with the same TE), before subsequently combining then, hence performing the NODDI fitting in normalised diffusion MRI instead of the raw images (Owen et al., 2014). However, we acknowledge that different T2 relaxation times in different compartments could still have an effect in our results (Veraart et al., 2017). 3) While the segmentation and parcellation of regions were performed with techniques specifically adapted to neonatal neuroimaging, we cannot discount that partial volume effects are still present in the region-wise estimation of microstructural features. To minimise this, we considered only median values, which are robust to outliers, and discarded most of possible cerebrospinal fluid contamination by introducing an additional threshold of f_iso_<0.5 to consider a voxel as part of cortical grey matter. 4) The different SNR of the 8-channel versus the 32-channel coil will have an impact on the diffusion measures obtained. We have included this as a co-factor in all statistical analyses, and visualising whole-brain grey matter tissue parameters discriminating by coil show similar patterns for both coils (Supplementary Fig. S11). 5) Our study is cross-sectional, while ideally longitudinal studies are required to assess maturation. 6) While we excluded preterm infants with major focal lesions on conventional MRI, infants who are born preterm are at-risk of impaired neurodevelopment (Johnson and Marlow, 2017; Moore et al., 2012). Previous studies have demonstrated a relationship between impaired cortical microstructural (Ball et al., 2013) and macrostructural development (Dubois et al., 2008a; Kapellou et al., 2006; Kersbergen et al., 2016; Rathbone et al., 2011) and subsequent neurodevelopmental performance. We do not have detailed neurodevelopmental assessments on the children in this study, but they are enrolled into long-term studies where we will assess the relationship between NODDI cortical measures and neurodevelopmental performance at school-age and beyond.

## 5 Conclusion

In summary, we observed a pattern of cortical maturation where cortical volume and curvature evolved in parallel with dendritic arborisation rather than with increases in cortical cellular density. Our findings are consistent with regional heterogeneity of microstructural maturation in the developing brain, where somatosensory areas develop first and association areas show a more protracted pattern of development. Overall, our results show the potential of structural and dMRI to characterise morphological and microstructural cortical development *in vivo*, which offers the opportunity to detect patterns of atypical maturation that lead to altered neurodevelopment.

## Funding

This work was supported by the Wellcome EPSRC Centre for Medical Engineering at King's College London (WT 203148/Z/16/Z), the Medical Research Council [grant numbers MR/K006355/1 and MR/L011530/1], the Department of Health through an NIHR Comprehensive Biomedical Research Centre Award (to Guy's and St. Thomas' National Health Service (NHS) Foundation Trust in partnership with King's College London and King's College Hospital NHS Foundation Trust), the National Institute for Health Research (NIHR) under its Programme Grants for Applied Research Programme [grant number RP-PG-0707-10154]. JOM is supported by a Sir Henry Dale Fellowship jointly funded by the Wellcome Trust and the Royal Society (grant number 206675/Z/17/Z). CK is supported by a British Heart Foundation Clinical Research Training Fellowship (grant number FS/15/55/31649)
